# Management of Implantable Cardioverter-Defibrillators during Pregnancy—A Systematic Review

**DOI:** 10.3390/jcm10081675

**Published:** 2021-04-14

**Authors:** Albert Topf, Nina Bacher, Kristen Kopp, Moritz Mirna, Robert Larbig, Mathias C. Brandt, Johannes Kraus, Uta C. Hoppe, Lukas J. Motloch, Michael Lichtenauer

**Affiliations:** 1 Department of Internal Medicine II, Paracelsus Medical University Salzburg, 5020 Salzburg, Austria; n.bacher@salk.at (N.B.); k.kopp@salk.at (K.K.); m.mirna@salk.at (M.M.); m.brandt@salk.at (M.C.B.); j.kraus@salk.at (J.K.); u.hoppe@salk.at (U.C.H.); l.motloch@salk.at (L.J.M.); m.lichtenauer@salk.at (M.L.); 2 Division of Cardiology, Hospital Maria Hilf Mönchengladbach, 41063 Mönchengladbach, Germany; robert.larbig@mariahilf.de; 3 Division of Electrophysiology, Department of Cardiovascular Medicine, University of Münster, 48149 Münster, Germany

**Keywords:** management, pregnancy, ICD, cardiomyopathy

## Abstract

Background: With the advent of implantable cardioverter-defibrillator (ICD) technology in recent decades, patients with inherited or congenital cardiomyopathy have a greater chance of survival into adulthood. Women with ICDs in this group are now more likely to reach reproductive age. However, pregnancy represents a challenge for clinicians, as no guidelines for the treatment of pregnant women with an ICD are currently available. Methods: To analyze this issue, we performed a systematic screening of the literature using the keywords: pregnancy with ICD, lead fracture in pregnancy, lead thrombi in pregnancy, ventricular tachycardia in pregnancy, inappropriate shocks in pregnancy, ICD discharge in pregnancy and ICD shock in pregnancy. Of 1101 publications found, 27 publications were eligible for further analysis (four retrospective trials and 23 case reports). Results: According to physiological changes in pregnancy, resulting in an increase in heart rate and cardiac output, a vulnerability for malignant arrhythmias and device-related complications in ICD carriers might be suspected. While the literature is limited on this issue, maternal complications including arrhythmia burden with following ICD therapies, thromboembolic events and lead complications as well as inappropriate shock therapy have been reported. According to the limited available studies, associated risk seems not to be more frequent than in the general population and depends on the underlying cardiac pathology. Furthermore, worsening of heart failure and related cardiovascular disease have been reported with associated risk of preterm delivery. These observations are exaggerated by restricted applications of diagnostics and treatment due to the risk of fetal harm in this population. Conclusions: Due to limited data on management of ICDs during pregnancy, further scientific investigations are required. Consequently, careful risk assessment with individual risk evaluation and close follow ups with interdisciplinary treatment are recommended in pregnant ICD carriers.

## 1. Introduction

Cardiac diseases, including arrhythmic disorders, are found in approximately one percent of all pregnancies [1]. These patients often require implantable cardioverter defibrillator (ICD) therapy due to high risk of sudden cardiac death (SCD). Although the majority of ICD implantations are undertaken in patients with acquired heart disease, the indications for an ICD implantation have been expanded to include younger age groups, in particular those with inherited and congenital heart disorders at risk of SCD [2]. Nevertheless, valid data on the prevalence of pregnant women with an ICD, wearable cardioverter defibrillator (WCD) or pacemaker are not available in the literature. In recent years, the number of patients carrying an ICD due to inherited or congenital cardiomyopathy has increased [3]. The main reasons for ICD implantations are structural heart diseases, including dilated cardiomyopathy (DCM) or hypertrophic cardiomyopathy (HCM), and inherited arrhythmogenic diseases such as long QT syndrome [2]. For younger female cardiac disease patients, survival to reproductive age with the desire of pregnancy is therefore becoming more common [4]. However, little is known regarding the outcome of pregnancy in women with ICDs as well as associated hemodynamic and electrophysiological changes in pregnancy. Indeed, physiological changes associated with pregnancy might trigger cardiac pathologies and, therefore, arrhythmia burden in these patients.

According to an ESC registry of 2966 pregnancies in women with structural heart disease, ventricular tachyarrhythmia (VTA) occurred in 1.4% of pregnant women, mainly in the third trimester [5]. Furthermore, VTA in pregnancy was associated with heart failure and had a clear impact on fetal outcome. New York Heart Association (NYHA) class before pregnancy was predictive for the prognosis [6]. The incidence of VTAs was 1.2% in patients with congenital heart defects, 0.6% in patients with valvular heart disease, 5.9% in cardiomyopathy patients, 2.1% in ischemic heart disease patients and 3% in patients with aortic pathologies. VTA was not observed in patients with pulmonary hypertension [7].

However, guidelines for the treatment of pregnant women with an ICD are still lacking. Published data with respect to the outcome of pregnancy in patients with an ICD are limited. Recommendations currently rely on case reports and retrospective studies.

In addition, further considerations should be applied when dealing with inherited but also acquired cardiac arrhythmogenic pathologies.

According to recommendations, inherited arrhythmia syndromes, including long QT syndrome (LQTS), catecholaminergic polymorphic ventricular tachycardia (CPVT), Brugada syndrome (BrS), short QT syndrome (SQTS), early repolarization syndrome (ERS) and arrhythmogenic cardiomyopathy (ACM), do not represent an absolute contraindication to pregnancy [8]. However, there is an increase in the risk of cardiac events in women with congenital LQTS, especially in the post-partum period [9].

The incidence of HCM in pregnancy is <1/1000 [10] and women with HCM usually tolerate pregnancy well. Maternal mortality is reported to be 0.5% and worsening of symptoms occurred in 29% of cases. Fetal mortality by spontaneous abortion, therapeutic abortion or stillbirth is comparable to the general population, however, the risk of premature birth is increased [11]. In particular, symptomatic women with a severe left ventricular outflow tract obstruction or a high prevalence of arrhythmia pre-pregnancy have a high risk of premature birth [12]. Beta-blockers should be continued or even started if symptoms occur. Hypovolemia is poorly tolerated. Low risk cases may have a spontaneous labor and vaginal delivery. Caesarean section should be considered with severe left ventricular outflow tract obstruction or severe heart failure. Epidural and spinal anesthesia must be applied cautiously due to potential hypovolemia [13].

Pregnancy is poorly tolerated in some patients with pre-existing DCM, with the potential of deterioration in left ventricular (LV) function. Predictors of maternal mortality are NYHA class III/IV and ejection fraction (EF) below 40%. All patients with DCM need a multidisciplinary care because of a high risk of irreversible deterioration of LV function, maternal mortality and fetal loss [14]. Patients with EF < 20%, right ventricular heart failure and hypotension are at especially high risk of adverse events [15]. Prior to conception, heart failure medications, such as angiotensin converting enzyme (ACE) inhibitors, angiotensin receptor II blockers (ARBs), angiotensin receptor II-blocker/neprolysin inhibitor (ARNI), mineralocorticoid receptor antagonists (MRAs) and ivabradine should be stopped to avoid fetal harm. Beta-blockers should, however, be continued with preference to beta-1-selective blockers [16]. If LV-function deteriorates, a discussion regarding the safety of pregnancy should be led. In stable congestive heart failure, vaginal delivery is preferred with spinal or epidural analgesia. Urgent delivery should be considered in women with advanced heart failure (HF) and hemodynamic instability despite treatment, irrespective of gestational duration. Caesarean section is recommended with central neuraxial anesthesia to prevent abrupt volume changes [17].

Despite the described findings and recommendations, in the vulnerable population of pregnant ICD patients, systematic analyses of preexisting arrhythmic risks and possible complications are still missing.

Therefore, the purpose of this systematic review was to describe the most commonly reported pregnancy-related risks. In a systematic analysis, we highlight the most important maternal, fetal, as well as device-related complications. Furthermore, a literature analysis of antiarrhythmic therapy options in pregnancy is also discussed.

To the best of our knowledge, this is the first review analyzing both complications during pregnancy as well as the outcome of pregnancy in patients with implanted ICD.

## 2. Materials and Methods

**Figure 1:**
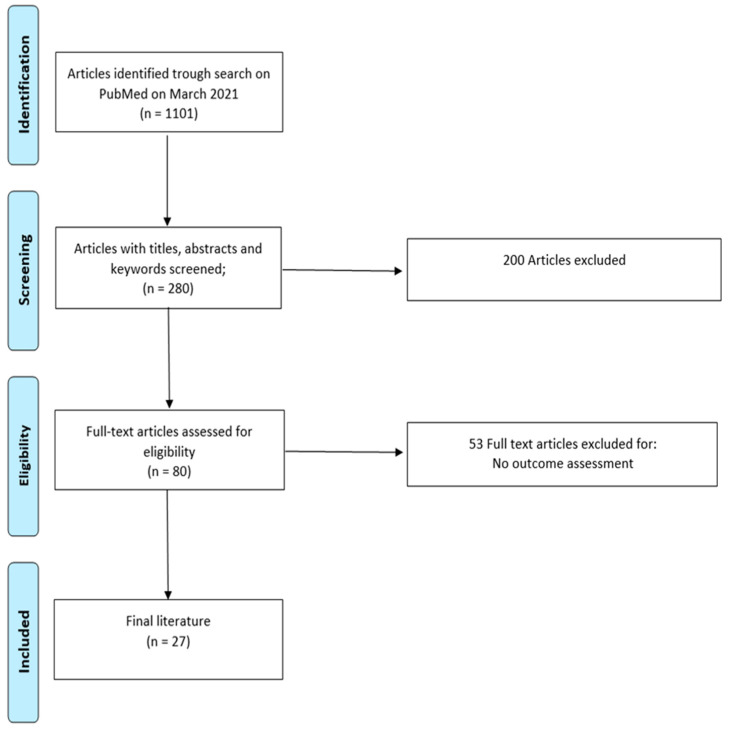
Flowchart outlining the protocol adopted in this systematic review for literature selection.

A systematic database search was conducted in the PubMed database between April 2020 and March 2021, using the terms ICD and pregnancy, lead fracture in pregnancy, lead thrombi in pregnancy, ventricular tachycardia in pregnancy, inappropriate shocks in pregnancy, ICD discharge in pregnancy and ICD shock in pregnancy. The authors screened all available studies (*n* = 1101) by title, and, if suitable, by abstract (*n* = 280). Duplicate manuscripts were excluded. At abstract level, 200 articles were excluded due to ineligibility. Figure 1 shows the search strategy results. At full-text level, we excluded studies (*n* = 53) that did not assess our outcome of interest. In total, four retrospective studies were included in the review. Additionally, available 23 case reports dealing with the given search terms were used for the creation of the manuscript.

## 3. Results

In systematic database search, using the terms ICD and pregnancy, lead fracture in pregnancy, lead thrombi in pregnancy, ventricular tachycardia in pregnancy, inappropriate shocks in pregnancy, ICD discharge in pregnancy and ICD shock in pregnancy, we found four eligible retrospective studies (Table 1), 22 case reports (Table 2) and one subgroup analysis of a study (Table 3) investigating the maternal, as well as the fetal outcome and the risk of device-related complications in pregnancy with an ICD. By reading the abstract and the full text, we excluded studies that did not assess our outcome of interest. Of these, the largest study included 44 patients [18], the smallest six patients [19] (Table 1). In four existing retrospective analyses, ICD carriers were pregnant at an age varying from 25.6 to 33 years [18,19,20,21]. In three out of four studies, the investigated women had been ICD carriers for at least one year before pregnancy [19,20,21]. In 25% to 64% of cases, an ICD was implanted for primary prophylaxis, and in 36% to 75% of cases for secondary prophylaxis.

The most common reasons for ICD implantation were DCM, idiopathic ventricular fibrillation and long QT syndrome (Table 4) [18,19,20,21].

### 3.1. Maternal Complications

#### 3.1.1. Deterioration of Heart Failure

Boulé et al. reported two patients suffering cardiac events after delivery. One patient experienced chest pain with a rise in troponin levels with subsequent transient reduction in LV ejection fraction with an immediate improvement after two months of ACE-inhibition (Table 5). Another patient with Tetralogy of Fallot was admitted with progressive right ventricular dilatation and exacerbation of pre-existing pulmonary regurgitation, requiring pulmonary valve replacement [20]. Schuler et al. described a heart failure patient with non-obstructive hypertrophic cardiomyopathy which worsened during pregnancy. However, reduced left ventricular function known prior to pregnancy remained stable throughout pregnancy and the response to diuretic therapy was satisfactory. An uncomplicated, induced vaginal delivery was possible at gestational age of 37 weeks [21]. In addition, Natale et al. reported one patient with a history of dilated cardiomyopathy who developed congestive heart failure with a reduction in LV-function during pregnancy, all of which resolved after delivery [18].

#### 3.1.2. Tachyarrhythmias with Consequent ICD Therapy

In the study by Natale et al., 10 out of 44 patients received a shock during pregnancy and after birth (Table 6). One patient experienced an inappropriate shock due to new onset atrial fibrillation, while the other patients received a shock in response to monomorphic ventricular tachycardia due to underlying severe coronary artery disease or dilated cardiomyopathy. During delivery, no shocks were observed [18]. Boulé et al. reported that one patient without an underlying structural heart disease, who primarily was not carrying an ICD, suffered an out of hospital cardiac arrest. Nine transthoracic defibrillations were necessary to control the electric storm. An ICD implantation at 6 weeks of gestation with following defibrillation testing was performed. Another patient received an appropriate shock at 4 weeks of gestation due to ventricular fibrillation. In a second instance, this patient was inappropriately shocked following sinustachycardia because of T wave oversensing by the ICD [20].

One patient with pre-existing LQTS in the study of Schuler et al. was reported to receive an appropriate shock due to ventricular fibrillation [21].

In addition to the two reports of inappropriate shocks in the retrospective studies, two case reports by Piper et al. and Olufolabi et al. described further inappropriate shock events. The patient in the case report by Olufolabi experienced an inadequate shock due to the onset of a SVT with similarity to preceding ventricular events. The problem could be pharmacologically solved [36]. Similarly, in the report by Piper et al., an onset of a supraventricular tachyarrhythmia triggered an inappropriate defibrillator discharge [38]. Further case reports describing 51 pregnancies in ICD patients did not report ICD discharges [22,34].

There were no reports of maternal deaths in ICD carriers during pregnancy in four retrospective studies or in any of the 23 case reports [31,35].

### 3.2. Fetal and Neonatal Complications

#### 3.2.1. Miscarriage and Still Birth

While Boulé reported a live birth rate of 14 out of 20 births in twelve patients [20], the live birth rate reported in the other studies was higher [18,19,21]. An analysis of the study by Boulé et al. revealed that one patient in the small population of twelve persons had three miscarriages, one woman had a stillbirth, one miscarriage was doubtfully associated with a preceding shock and one pregnancy was terminated at 15 weeks gestation due to maternal danger following a heart surgery [20]. In the study by Schuler et al., one pregnancy was terminated due to a detected, severe, fetal chromosomal abnormality at 10 weeks gestation age [21]. Natale et al. reported one stillbirth due to cord strangulation [18].

Adverse fetal outcomes following the occurrence of ICD shocks were not described in the literature, with the exception of one report of ICD shock during the early stage of pregnancy [20]. Ventricular fibrillation in one pregnancy with a subsequent ICD shock and an inadequate shock following a sinus tachycardia with T wave oversensing might have resulted in a miscarriage seven days later [20], but this constellation will need further investigation in future studies. Natale et al. reported that among eleven women experiencing shocks, ten infants were born healthy and one had transient hypoglycemia, which was attributed to the mother’s sotalol therapy [18]. An ICD shock at 20 weeks of gestation in a patient with LQTS in the study by Schuler et al. remained without adverse fetal outcome [21]. Further doubt on the relationship between an ICD shock and a subsequent miscarriage is given, as the following reports describe ICD discharges occurring during first trimester of pregnancy without adverse outcomes. Bonini et al. reported an ICD discharge at 10 weeks’ gestation due to ventricular fibrillation without any adverse fetal outcomes [26]. In a report of Ahmed et al., a woman with a confirmed CPVT received three shocks and experienced several episodes of antitachycardia pacing during the first trimester and gave birth to a healthy newborn with an average birth weight [28]. Another woman in the report by Hodes et al., experienced an ICD discharge at three weeks gestation and the newborn showed no abnormalities [43]. In a dramatic report by Burrows et al., a patient received 200 discharges in eight days, but nevertheless, she gave birth to an infant at 24 weeks, who could be discharged home at a full term corrected gestational age [27].

#### 3.2.2. Preterm Delivery

With respect to preterm delivery, Schuler et al. [21] observed that, in three out of 19 pregnancies, delivery prior to 36 weeks was necessary due to symptomatic palpitations and left ventricular failure (Table 7). Francia presented a case report of a woman with hypertrophic cardiomyopathy with the need for preterm delivery due to progressive worsening of heart failure symptoms [39]. In the report by Mitsui, a woman with a severe HOCM at gestation week 27 had to undergo a preterm delivery due to a progression and exacerbation of her HOCM with pulmonary edema during pregnancy [32]. However, no cases of preterm delivery associated with ICD shock had been reported so far.

### 3.3. Device-Related Complications

#### 3.3.1. Thrombotic Complications

Schuler et al. presented one case of a lead thrombus in a pregnant woman with HOCM and an ICD for primary prevention of sudden cardiac death, but good biventricular function was maintained. The thrombus with a size of 13 × 15 mm attached to the ventricular lead in close proximity to the tricuspid valve was identified in the second trimester. Thrombophilia screening was conducted and a homozygous polymorphism for factor V was found. Furthermore, family history of thrombotic events was reported. Anticoagulation with Dalteparin was administered and the ICD system was replaced in this patient. The patient made an uneventful recovery and underwent elective caesarean section at 36 weeks gestation [21]. In further literature research, including 23 case reports, no lead thrombi were described [25]. Natale et al. described a case of pulmonary embolism during pregnancy [18].

#### 3.3.2. Lead Failure and/or Fracture

In the literature, two cases of lead fracture were reported (Table 8). In the study by Schuler et al., a pacemaker-dependent patient with HCM and a previous Ross surgery developed high atrial impedance in the second trimester and further investigation showed an atrial lead fracture [21]. Al-Aqeedi et al. reported of a case of multigravida, in which the patient needed a revision of a six-year-old ventricular shock lead after the delivery of her second child [40].

### 3.4. Delivery

So far, no appropriate or inappropriate ICD shocks during delivery have been reported in the literature (see Table 9). Furthermore, as already reported above, in contrast to transthoracic shock therapy, the fetal risk during ICD shock seems low at labor because of a limited transferred energy to the uterus when ICD firing occurs [20]. Therefore, in reported studies, antitachycardia therapy remained on during vaginal deliveries as well as during cesarean sections (C-sections) as long as the cautery was not involved.

### 3.5. Sucbcutaneous ICD and Wearable Cardioverter/Defibrillator in Pregnancy

Subcutaneous ICD (S-ICD) systems emerge as an alternative system for the prevention of SCD. Particularly in younger patients with a need for longevity of the system, S-ICD systems gain more importance in the guidelines. Implantation of an S-ICD, if an ICD indication emerges during pregnancy, should even be considered in order to avoid fluoroscopy [9]. Nevertheless, the literature on S-ICD in pregnancy is limited to a case report. During delivery, the S-ICD was deactivated [42].

With the exception of one case report of Reuschel et al. of a woman provided with a wearable cardioverter/defibrillator (WCD) during pregnancy due to refusal of a permanent ICD system, the literature of WCD during pregnancy is limited to reports of women with peripartal cardiomyopathy (PPCM) [44]. In a single center observational study of Dunker et al., seven patients with PPCM were provided with a WCD. Three of those seven women had an appropriate and successful discharge after delivery and no woman had died as long as provided with a WCD [45]. In a study of Saltzberg, 107 women with PPCM were enrolled. No shocks or lethal outcomes had been observed for the time of supply with a WCD [46].

### 3.6. The Management of Antiarrhythmic Therapy

The most common way to treat tachyarrhythmia in pregnancy is to initiate antiarrhythmic drugs. Modifications on the programming of ICDs are only reported, if malfunction, like T-wave oversensing, requires a reprogramming. [20] The initiation of antiarrhythmic therapy in pregnancy has to be carefully assessed with consideration of therapeutic benefits and potential fetal as well as maternal risks of antiarrhythmic drugs. Recommendations for antiarrhythmic regime in pregnancy have to be respected [47].

Beta-blockers play an important role in the antiarrhythmic treatment of ventricular tachycardia during pregnancy and while breastfeeding, especially for long QT syndrome or CPVT [48]. All beta-blockers have the potential to affect fetal and newborn growth, but only atenolol has been singled out as being a Food and Drug Administration (FDA) class D drug. The rest are FDA class B or C [49]. In addition to beta-blockers, verapamil is recommended in the European guidelines for pregnancy and arrhythmias in the long-term therapy for prevention of idiopathic sustained VT [9].

Sotalol and intravenous procainamide can be used to convert hemodynamically stable monomorphic VTs. If an unstable monomorphic VT is not responding to cardioversion or the aforementioned drugs, amiodarone can be administered [50].

## 4. Discussion

Altogether, the literature is limited to 133 pregnant women with an ICD. With the exception of one pregnant woman, all patients have been ICD carriers prior to pregnancy. When mentioned in the literature, the indication for ICD implantation was predominantly for secondary prevention of SCD (see Table 1). Women with an ICD at child bearing age belong to a highly vulnerable group. Because of advanced, complex underlying cardiac diseases, women with certain cardiologic entities are at risk of an exacerbation of their disease during pregnancy and are generally advised not become pregnant [10].

Women with structural heart diseases are especially jeopardized because pregnancy might deteriorate pre-existing cardiac conditions. Therefore, careful interrogation prior to pregnancy with individual risk evaluation is necessary [51].

Pregnancy is expected to be accompanied by proarrhythmic risk due to physiological changes during pregnancy or deterioration of preceding cardiac diseases. Pregnant women have a higher risk of experiencing supraventricular tachycardias. The most common type is a reentrant tachycardia [52]. Patients with a preceding electrical disorder or a structural, cardiac disease may be expected to have also an increased number of ventricular arrhythmias or more frequent ICD firings due to hemodynamic changes or/and autonomic nervous system alterations during pregnancy [18]. Reports of either appropriate or inappropriate ICD discharges are listed in Table 2 and Table 6. Adverse fetal outcomes following the occurrence of ICD shocks or antitachycardia pacing are not found in the literature, with the exception of one report of an ICD shock during the early stage of pregnancy. There is doubt as to the relation, as other reports at early gestational age, with shocks at three weeks gestation age and multiple shocks at early gestation, remained uneventful. As idiopathic miscarriage occurs in 15% of the background population, the case of miscarriage, described by Miyoshi et al., might not be related to the preceding ICD discharge [19]. In contrast to ICD shocks, transthoracic shocks were reported to result in severe, sustained fetal bradycardia [53]. It has been hypothesized that the uterus is a likely good conductor of electricity and contracts following transthoracic shocks. ICD shocks, however, are targeted away from the uterus [20]. Another reason for the lack of adverse fetal side events is that the fetal myocardium has a high fibrillation threshold and that low transmission of shock energy is conducted to the fetus [54]. Based on this observation, and the fact that no ICD firings have been described during delivery in the literature, it is recommended that antitachycardia function remains on during vaginal deliveries as well as during cesarean sections (C-sections) as long as the cautery is not involved [18]. According to a prevalence of 13% of C-sections in the general population, operative delivery seems more common in the evaluated studies of pregnant women with an ICD [55].

In patients with an underlying cardiac disease, the rate of miscarriage is even reported to be 12–24%. With regard to cardiac patients, the presence of maternal cyanosis and reduced cardiac output are known predictors of fetal growth restriction and might result in miscarriage [56]. Most of the miscarriages were reported in a small retrospective analysis that included twelve women. Among the patients with reported miscarriage, one woman suffered three miscarriages, another woman had a stillbirth without specified circumstances and one miscarriage was due to cord strangulation. One pregnancy had to be terminated due to maternal danger and another one due to severe, fetal chromosomal abnormality [18,20,21].

As aforementioned, preterm delivery was solely associated with exacerbation of preexisting structural heart diseases [57]. However, no cases of preterm delivery were referred to ICD shocks. This observation emphasizes the relevance of individual risk evaluation and close follow ups with interdisciplinary treatment.

The most relevant device-related complications were thrombus formations on leads and the risk of lead fractures. In the literature, one case of a lead thrombus was described. In a study from 2003, Chow et al. describe an incidence of 25% of lead thrombi in the general device carrier population, independent of pregnancy. Of note, in the study of Chow et al., one patient out of 46 patients with diagnosed lead thrombi developed symptomatic pulmonary embolism. However, most thrombi were small and subclinical [58,59]. Another thrombotic event with a pulmonary embolism was reported in the literature of pregnant women. However, the described prevalence of thromboembolic events in patients with an ICD might be in accordance with the average incidence of 0.1–0.5% in pregnancy [60]. The risk of a lead fracture is a major concern. Due to fetal growth, the diaphragm elevates, abdominal girth expands, and contractions associated with labor may increase stress on the transvenous lead system, which potentially may lead to risk of a lead fracture. Lead complications are difficult and dangerous to treat in pregnancy. Lead explantation and re-implantation are associated with elevated procedural risk [19]. In the literature, two cases of lead fracture were reported. Nevertheless, with an incidence of 2.6 to 3.6% of lead fractures in the average population, the reported device complications seem comparable to the standard population [61].

## 5. Conclusions and Limitations

The number of female ICD carriers reaching childbearing age with the desire to have a baby is expected to increase. Despite this, systematic guidelines to treat those patients are missing. Recommendations are restricted to limited literature. According to physiological changes in pregnancy, resulting in an increase in heart rate and cardiac output, a vulnerability for malignant arrhythmia might be suspected. However, compared to other ICD carriers and the general population, an increased rate of ICD therapy and miscarriages have not been described yet. Nevertheless, data are limited on this subject. Therefore, further investigations are necessary to assess this issue. A similar risk seems to be evident for device-related complications. However, inappropriate shocks have also been described. Furthermore, pregnancy can exacerbate preexisting cardiac conditions, resulting in a worsening of underlying cardiac diseases and a consequent increased risk of preterm delivery dependent on the underlying cardiac pathology. In addition, it might hamper diagnostics and treatment of underlying arrhythmic pathologies due to the risk of fetal harm. Thus, precise interrogation prior to pregnancy with individual risk evaluation, and close follow ups with interdisciplinary treatment during pregnancy, are necessary in this vulnerable patient group. Further large, prospective studies are necessary to determine guidelines for pregnancy and delivery in patients with an ICD.

## Figures and Tables

**Table 1:** Overview of the four retrospective studies describing pregnancy with an implantable cardioverter defibrillator (ICD). The age of pregnancy, the duration of ICD carriage before pregnancy, the reason for implantation (primary vs. secondary prevention of sudden cardiac death), the number of people taking antiarrhythmics and the left ventricular ejection fraction (LVEF) is given.

Study	*n*	Age	Carrier (years)	Primary Implant. (*n*)	Secondary Implant. (*n*)	Antiarrythmics (*n*)	LVEF (%)
Boulé	12	28 ± 5	-	3	9	9	>55
Miyoshi	6	25 ± 6	5 ± 3	-	-	6	53 ± 2
Schuler	14	33	3.8	9	5	12	>55
Natale	44	25.6 ± 4.9	4.8 ± 2.8	11	33	25	49.8 ± 9.7

**Table 2:** Summary of the case reports and case series, including the authors’ names, the number of patients presented (*n*) and the most important complications.

Authors	*n*	Complications
Bouslama et al. [22]	1	Patient with HOCM, no adverse events
Howell et al. [23]	1	Patient with non obstructive HCM, no adverse events
Kanniah et al. [24]	1	Patient with Holt–Oram syndrome, no adverse events
Luo et al. [25]	5	5 Patients with ARVC, no adverse events
Bonini et al. [26]	1	Patient with IVF, ICD discharge at 10 weeks gestation
Burrows et al. [27]	1	Patient with IVF, 200 discharges during pregnancy, infant was discharged at full-term corrected gestational age, mother needed intermitted venoarterial extracorporeal membrane oxygenation, discharged on hospital day 19
Ahmed et al. [28]	1	Patient with CPVT, 3 ICD discharges at 2 weeks, 4 weeks and 9 weeks gestation, delivered a healthy male newborn
Rodríguez-Mañero et al. [29]	3	3 Patients with Brugada Syndrome, no discharges
Salman et al. [30]	2	1 Patient with LQTS and 1 Patient with HOCM, no ICD discharge
Schumer et al. [31]	1	1 Patient with CPVT, no ICD discharge, healthy newborn
Smeets et al. [7]	1	1 Patient with SCD and a Marfan Syndrome, no discharge, no adverse events
Mitsui et al. [32]	1	Patient with HOCM, preterm delivery due to worsening of cardiomyopathy with pulmonary congestion
Michalak et al. [33]	1	Patient with IVF, Pregnancy without complications
Al-Refai et al. [34]	1	Patient with LQTS, no adverse events
Doyle et al. [35]	1	Patient with ARVC, no adverse events
Olufolabi et al. [36]	1	Patient with repetitive episodes of sustained VT, 1 appropriate shock at 18 weeks gestation and 1 inappropriate shock at 3rd trimester
Knops et al. [37]	1	Patient with IVF, no ICD discharge during pregnancy
Piper et al. [38]	1	Healthy newborn despite an inappropriate discharge at 33 weeks gestation due to atrial fibrillation
Francia et al. [39]	2	2 patients with non-obstructive HCM, no ICD discharge, preterm delivery due to worsening of the cardiomyopathy in 1 patient
Al-Aqeedi et al. [40]	1	ICD in DCMP, extraction and reimplantation of a 6 year old shock lead after her second delivery
Ergle et al. [41]	1	ICD to VT because of recurring coronary spasm, ICD discharge at 17 weeks gestation, preterm delivery at 24 weeks due to electrical storm
Strewe et al. [42]	1	One woman with PPCM and S-ICD, deactivated during pregnancy

**Table 3:** Subgroup analysis of the ICD population in a study investigating the maternal and fetal outcome of pregnant women with an underlying ARVC.

Authors	*n*	Complications
Hodes et al. [43]	28	28 patients with ARVC, live birth despite ICD discharge at 3 weeks gestation in one patient

**Table 4:** The underlying cardiac disease for ICD implantation.

Study	ToF (*n*)	HCM (*N*)	LQTS (*N*)	DCM (*N*)	IVF (*N*)	ARVC (*N*)	CPVT (*N*)	LPS (*N*)	CHD (*N*)
Boulé	1	2	1	0	3	2	1	1	1
Miyoshi	0	0	2	2	1	0	0	0	1
Schuler	1	8	3	1	1	0	0	0	0
Natale	0	1	13	9	17	1	0	0	3

ToF (Tetralogy of Fallot). HCM (hypertrophic cardiomyopathy). LQTS (long QT syndrome). DCM (dilatative cardiomyopathy). IVF (idiopathic ventricular fibrillation). ARVC (arrythmogenic right ventricular cardiomyopathy). CPVT (catecholaminergic polymorphic ventricular tachycardia). LPS (Laubry–Pezzi syndrome). CHD (congenital heart disease).

**Table 5:** Maternal complications of pregnancy with an ICD.

Study	Death (*n*)	Heart Failure (*n*)	Thrombosis/Thromboembolism (*n*) and %	VT/VF (*n*)	SVT (*n*)	Worsening LVEF (*n*)	Valve Replacement (*n*)
Boulé	0	0	0	2	0	1	1
Miyoshi	0	0	0	3	1	1	0
Schuler	0	1	1	3	1	0	0
Natale	0	1	1	10	1	0	0

**Table 6:** Highlight the risk of appropriate and inappropriate shocks in pregnant women with an ICD.

Study	*n*	No Discharge (*n*)	Shock (*n*)	Inappropiate Shock (*n*)
Boulé	12	10	2	1
Miyoshi	6	6	0	0
Schuler	14	13	1	0
Natale	44	33	10	1

**Table 7:** The number of live births, preterm delivery/labor, the Apgar score at 5 and 10 min, the mean birth weight in grams, the number of fetuses with lower birth weight (LBW), intrauterine growth retardation (IUGR) and neonatal hypoglycemia (NHG).

Study	Pregnancies	Live Birth	Preterm Delivery/Labor	Apgar Score 5 min	Apgar Score 10 min	Mean Birth Weight (g)	LBW	IUGR	NHG
Boulé	20	14	5	10	10	2690 ± 596	3	4	5
Miyoshi	6	6	2	8	9.33	2210 ± 603	3	3	3
Schuler	19	18	3	-	-	2900	-	-	-
Natale	40	39	-	-	-	-	2	2	1

**Table 8:** Device-related complications.

Study	*n*	Lead Thrombus (*n*)	Lead fracture (*n*)
Boulé	12	0	0
Miyoshi	6	0	0
Schuler	14	1	1
Natale	44	0	0

**Table 9:** The mean gestation age, delivery method, occurrence of ventricular arrhythmia, number of shocks during delivery and activation status of the ICD during delivery, are presented.

Study	Mean Gestation Age (Weeks)	Vaginal Delivery	Caesarean Section	Arrythmia	Shock	Antitachycardia Therapy: On
Boulé	37	6	6	No	No	-
Miyoshi	37 + 2	0	6	No	No	0
Schuler	38	12	5	No	No	-
Natale	-	37	7	No	No	28

## Data Availability

Not applicable.

## References

[1] Hollier L.M., James M.D., Martin N., Heidi M.D., Connolly M.D., Turrentine M., Afshan M.D. (2019). ACOG Practice Bulletin No. 212: Pregnancy and Heart Disease. Obstet. Gynecol..

[2] Ishikawa T. (2013). Implantable cardioverter defibrillator therapy during pregnancy—Is it safe and effective?. Circ. J..

[3] Goldstein S., Ward C., Al-Khatib S. (2018). The Use of Implantable Cardioverter-defibrillators in the Prevention of Sudden Cardiac Death: A Focus on Congenital Heart Disease and Inherited Arrhythmia Syndromes. J. Innov. Card. Rhythm Manag..

[4] Phillips S., Pirics M. (2017). Congenital Heart Disease and Reproductive Risk: An Overview for Obstetricians, Cardiologists, and Primary Care Providers. Methodist DeBakey Cardiovasc. J..

[5] Ertekin E., van Hagen I.M., Salam A.M., Ruys T.P., Johnson M.R., Popelova J., Parsonage W.A., Ashour Z., Shotan A., Oliver J.M. (2016). Ventricular tachyarrhythmia during pregnancy in women with heart disease: Data from the ROPAC, a registry from the European Society of Cardiology. Int. J. Cardiol..

[6] Grewal J., Siu S.C., Ross H.J., Mason J., Balint O.H., Sermer M., Colman J.M., Silversides C.K. (2009). Pregnancy Outcomes in Women with Dilated Cardiomyopathy. J. Am. Coll. Cardiol..

[7] Smeets C.J., Vranken J., Van Der Auwera J., Verbrugge F.H., Mullens W., Dupont M., Grieten L., De Cannière H., Lanssens D., Vandenberk T. (2017). Bioimpedance Alerts from Cardiovascular Implantable Electronic Devices: Observational Study of Diagnostic Relevance and Clinical Outcomes. J. Med. Internet Res..

[8] Conte G., Scherr D., Lenarczyk R., Gandjbachkh E., Boulé S., Spartalis M.D., Behr E.R., Wilde A., Potpara T. (2020). Diagnosis, family screening, and treatment of inherited arrhythmogenic diseases in Europe: Results of the European Heart Rhythm Association Survey. EP Eur..

[9] Priori S.G., Blomström-Lundqvist C., Mazzanti A., Blom N., Borggrefe M., Camm J., Elliott P.M., Fitzsimons D., Hatala R., Hindricks G. (2015). 2015 ESC Guidelines for the management of patients with ventricular arrhythmias and the prevention of sudden cardiac Death. The Task Force for the Management of Patients with Ventricular Arrhythmias and the Prevention of Sudden Cardiac Death of the European Society of Cardiology. Eur. Heart J..

[10] Regitz-Zagrosek V., Roos-Hesselink J.W., Bauersachs J., Blomström-Lundqvist C., Cífková R., De Bonis M., Iung B., Johnson M.R., Kintscher U., Kranke P. (2018). 2018 ESC Guidelines for the management of cardiovascular diseases during pregnancy. Eur. Hearth J..

[11] Metra M. (2016). September 2016 at a glance: Pregnancy, hypertrophic cardiomyopathy, epidemiology, medical treatment. Eur. J. Hearth Fail..

[12] Tanaka H., Kamiya C., Katsuragi S., Tanaka K., Miyoshi T., Tsuritani M., Yoshida M., Iwanaga N., Neki R., Yoshimatsu J. (2014). Cardiovascular Events in Pregnancy with Hypertrophic Cardiomyopathy. Circ. J..

[13] Regitz-Zagrosek V., Lundqvist C.B., Borghi C., Cifkova R., Ferreira R., Foidart J.-M., Gibbs J.S.R., Gohlke-Baerwolf C., Gorenek B., Iung B. (2011). ESC Guidelines on the management of cardiovascular diseases during pregnancy: The Task Force on the Management of Cardiovascular Diseases during Pregnancy of the European Society of Cardiology (ESC). Eur. Hearth J..

[14] Schaufelberger M. (2019). Cardiomyopathy and pregnancy. Hearth.

[15] Mandal D., Mandal S., Mukherjee D., Biswas S.C., Maiti T.K., Chattopadhaya N., Majumdar B., Panja M. (2010). Pregnancy and subsequent pregnancy outcomes in peripartum cardiomyopathy. J. Obstet. Gynaecol. Res..

[16] Pieper P.G. (2011). The pregnant woman with heart disease: Management of pregnancy and delivery. Neth. Hearth J..

[17] Sliwa K., Anthony J. (2016). Decompensated Heart Failure in Pregnancy. Card. Fail. Rev..

[18] Natale A., Davidson T., Geiger M.J., Newby K. (1997). Implantable cardioverter-defibrillators and pregnancy: A safe combination?. Circulation.

[19] Miyoshi T., Kamiya C.A., Katsuragi S., Ueda H., Kobayashi Y., Horiuchi C., Yamanaka K., Neki R., Yoshimatsu J., Ikeda T. (2013). Safety and Efficacy of Implantable CardioverterDefibrillator During Pregnancy and After Delivery. Circ. J..

[20] Boulé S., Ovart L., Marquié C., Botcherby E., Klug D., Kouakam C., Brigadeau F., Guédon-Moreau L., Wissocque L., Meurice J. (2014). Pregnancy in women with an implantable cardioverter-defibrillator: Is it safe?. Europace.

[21] Schuler P.K., Herrey A., Wade A., Brooks R., Peebles D., Lambiase P., Walker F. (2012). Pregnancy outcome and management of women with an implantable cardioverter defibrillator: A single centre experience. Europace.

[22] Bouslama M.A., Ferhi F., Hacheni F., Ons K., Abdeljelil K., Ben Jazia K., Khairi H. (2018). Pregnancy and delivery in woman with implantable cardioverter-defibrillator: What we should know. Pan Afr. Med. J..

[23] Howell L.A., Schwartz J. (2015). ICD Management During Pregnancy and Delivery. J. Am. Coll. Cardiol..

[24] Kanniah S.K. (2008). Caesarean delivery in a parturient with Holt–Oram syndrome and implantable cardioverter defibrillator: Anaesthetic considerations. Arch. Gynecol. Obstet..

[25] Luo F.Y., Chadha R., Osborne C., Kealey A. (2020). Arrhythmogenic Right Ventricular Cardiomyopathy (ARVC) in pregnancy: A case series of nine patients and review of literature. J. Matern. Neonatal Med..

[26] Bonini W., Botto G.L., Broffoni T., Dondina C. (2000). Pregnancy with an ICD and a documented ICD discharge. Europace.

[27] Burrows K., Fox J., Biblo L.A., Roth J.A. (2010). Pregnancy and Short-Coupled Torsades de Pointes. Pacing Clin. Electrophysiol..

[28] Ahmed A., Phillips J.R. (2016). Teenage pregnancy with catecholaminergic polymorphic ventricular tachycardia and documented ICD discharges. Clin. Case Rep..

[29] Rodríguez-Mañero M., Casado-Arroyo R., Sarkozy A., Leysen E., Sieira J.A., Namdar M., Conte G., Levinstein M., Chierchia G.-B., De Asmundis C. (2014). The Clinical Significance of Pregnancy in Brugada Syndrome. Rev. Española Cardiol. (Engl. Ed.).

[30] Salman M., Kemp H., Cauldwell M., Dob D., Sutton R. (2018). Anaesthetic management of pregnant patients with cardiac implantable electronic devices: Case reports and review. Int. J. Obstet. Anesth..

[31] Schumer A., Contag S. (2020). Catecholaminergic polymorphic ventricular tachycardia in pregnancy: A case report. J. Med. Case Rep..

[32] Mitsui T., Masuyama H., Ejiri K., Hayata K., Ito H., Hiramatsu Y. (2016). A Pregnancy with Severe Hypertrophic Obstructive Cardiomyopathy after Surgery for an Implantable Cardioverter Defibrillator: A Case Report and Literature Review. Case Rep. Obstet. Gynecol..

[33] Michalak M., Cacko A., Grabowski M. (2014). Pregnancy-related physiological changes in cardiovascular system observed with implantable cardioverter-defibrillator. Kardiol. Pol..

[34] Al-Refai A., Gunka V., Douglas J. (2004). Spinal anesthesia for Cesarean section in a parturient with long QT syndrome. Can. J. Anaesth..

[35] Doyle N.M., Monga M., Montgomery B., Dougherty A.H. (2005). Arrhythmogenic right ventricular cardiomyopathy with implantable cardioverter defibrillator placement in pregnancy. J. Matern. Neonatal Med..

[36] Olufolabi A., Charlton G., Allen S., Mettam I., Roberts P. (2002). Use of implantable cardioverter defibrillator and anti-arrhythmic agents in a parturient. Br. J. Anaesth..

[37] Knops P., Jordaens L. (2008). Recurrence of cardiac arrest after 14 years without ICD interventions: A VF cluster immediately after delivery. Neth. Hearth J..

[38] Piper J.M., Berkus M., Ridgway L.E. (1992). Pregnancy complicated by chronic cardiomyopathy and an automatic implantable cardioverter defibrillator. Am. J. Obstet. Gynecol..

[39] Francia P., Adduci C., Musumeci B., Semprini L., Palano F., Zezza L., Volpe M., Autore C. (2018). Autonomic cardiovascular control and cardiac arrhythmia in two pregnant women with hypertrophic cardiomyopathy: Insights from ICD monitoring. Rev. Port. Cardiol..

[40] Al-Aqeedi R.F., Alnabti A., Al-Ani F., Dabdoob W., Abdullatef W.K. (2011). Successful delivery by a cesarean section in a parturient with severe dilated cardiomyopathy, an implantable cardioverter defibrillator, and a repaired tetralogy of fallot. Heart Views Off. J. Gulf Heart Assoc..

[41] Ergle K., Bernard M. (2019). Refractory Ventricular Tachycardia from Coronary Vasospasm During Pregnancy. Ochsner J..

[42] Strewe C., Fichtner S. (2015). Completely subcutaneous implantable cardioverter defibrillator: Care of S-ICD wearers during childbirth. Anaesthesist.

[43] Hodes A.R., Tichnell C., Riele A.S.J.M.T., Murray B., Groeneweg J.A., Sawant A.C., Russell S.D., Van Spaendonck-Zwarts K.Y., Berg M.P.V.D., Wilde A.A. (2015). Pregnancy course and outcomes in women with arrhythmogenic right ventricular cardiomyopathy. Hearth.

[44] Reuschel E., Baessler A., Stöllberger C., Finsterer J., Maier L., Fischer M., Poschenrieder F., Heissenhuber F., Kurzidim K., Schepp C. (2016). Interdisciplinary management of left ventricular hypertrabeculation/noncompaction during pregnancy with a wearable defibrillator. Int. J. Cardiol..

[45] Duncker D., Haghikia A., König T., Hohmann S., Gutleben K.-J., Westenfeld R., Oswald H., Klein H., Bauersachs J., Hilfiker-Kleiner D. (2014). Risk for ventricular fibrillation in peripartum cardiomyopathy with severely reduced left ventricular function-value of the wearable cardioverter/defibrillator. Eur. J. Hearth Fail..

[46] Saltzberg M.T., Szymkiewicz S., Bianco N.R. (2012). Characteristics and Outcomes of Peripartum Versus Nonperipartum Cardiomyopathy in Women Using a Wearable Cardiac Defibrillator. J. Card. Fail..

[47] Canobbio M.M., Warnes C.A., Aboulhosn J., Connolly H.M., Khanna A., Koos B.J., Mital S., Rose C., Silversides C., Stout K. (2017). Management of Pregnancy in Patients with Complex Congenital Heart Disease: A Scientific Statement for Healthcare Professionals From the American Heart Association. Circulation.

[48] Roston T.M., van der Werf C., Cheung C.C., Grewal J., Davies B., Wilde A.A., Krahn A.D. (2020). Caring for the pregnant woman with an inherited arrhythmia syndrome. Heart Rhythm.

[49] Duan L., Ng A., Chen W., Spencer H.T., Lee M.-S. (2018). Beta-blocker subtypes and risk of low birth weight in newborns. J. Clin. Hypertens..

[50] Adamson D.L., Nelson-Piercy C. (2007). Managing palpitations and arrhythmias during pregnancy. Heart.

[51] Adam K. (2017). Pregnancy in Women with Cardiovascular Diseases. Methodist DeBakey Cardiovasc. J..

[52] Robins K., Lyons G. (2004). Supraventricular tachycardia in pregnancy. Br. J. Anaesth..

[53] Batra A.S., Balaji S. (2019). Fetal arrhythmias: Diagnosis and management. Indian Pacing Electrophysiol. J..

[54] Merino J.L., Perez-Silva A. (2011). Tachyarrhythmias and pregnancy. E J. Cardiol. Pract..

[55] Karim F., Ali N.B., Khan A.N.S., Hassan A., Hasan M.M., Hoque D.M.E., Billah S.M., El Arifeen S., Chowdhury M.A.K. (2020). Prevalence and factors associated with caesarean section in four Hard-to-Reach areas of Bangladesh: Findings from a cross-sectional survey. PLoS ONE.

[56] Gelson E., Curry R., Gatzoulis M.A., Swan L., Lupton M., Steer P., Johnson M. (2011). Effect of Maternal Heart Disease on Fetal Growth. Obstet. Gynecol..

[57] Makino Y., Matsuda Y., Mitani M., Shinohara T., Matsui H. (2012). Risk factors associated with preterm delivery in women with cardiac disease. J. Cardiol..

[58] Chow B.J., Hassan A.H., Chan K.L., Tang A.S. (2003). Prevalence and significance of lead-related thrombi in patients with implantable cardioverter defibrillators. Am. J. Cardiol..

[59] Page R.L., Hamdan M.H., Joglar J.A. (2002). Arrhythmias occurring during pregnancy. Card. Electrophysiol. Rev..

[60] Zhu R., Hu Y., Tang L. (2017). Reduced cardiac function and risk of venous thromboembolism in Asian countries. Thromb. J..

[61] Sato D., Kitajima H., Mani H., Park C.-H., Chun Y.-H. (2013). Pacemaker lead fracture without an increase in lead impedance caused by cardiac fibroma. J. Arrhythmia.

